# A combination of herbal formulas, acupuncture, and novel pine-needle stimulation for recurrent alopecia areata: A case report

**DOI:** 10.1097/MD.0000000000026084

**Published:** 2021-05-21

**Authors:** Nozomu Kawashima, Xiaochen Hu, Nagako Ishikawa, Takaharu Matsuhisa, Juichi Sato

**Affiliations:** a Department of Pediatrics; b Department of General Medicine/Family & Community Medicine, Nagoya University Graduate School of Medicine, Nagoya, Japan.

**Keywords:** alopecia areata, formulas, japanese kampo medicine, pine-needle acupuncture, plum-blossom acupuncture

## Abstract

**Introduction:**

Head hair is a symbol of vitality, and hair loss by alopecia areata (AA) presents a burden on patients. Although traditional Japanese Kampo medicine (JKM) formulas, acupuncture, and moxibustion have historically been used for treating AA, no studies have utilized a combination of these modalities.

**Patient concerns:**

A 34-year-old male with a history of childhood asthma presented with a sudden hair loss at the top of his head without any preceding symptoms. Except for a hairless patch of 5 cm × 6 cm, his general appearance was otherwise good. The patient underwent topical immunotherapy on visiting a dermatologist. However, the patient noticed an exacerbation of his hairless lesion.

**Diagnosis:**

The AA diagnosis was established based on clinical appearance and dermatological findings. The Severity of Alopecia Tool (SALT) score for alopecia was 19% at diagnosis.

**Interventions:**

The patient received 2 JKM formulas (saikokaryukotsuboreito and shichimotsukokato) in combination with acupuncture. When relapse occurred, a novel self-administration of pine-needle acupuncture was initiated in combination with the JKM formulas.

**Outcomes:**

A 50% recovery from the baseline SALT score was achieved using JKM formulas in combination with acupuncture for 4 months. The patient achieved complete remission for 5 months. However, another stressful event induced an AA relapse with multiple lesions harboring a SALT score of 13%. Pine-needle acupuncture was initiated, resulting in faster resolution than the first treatment. Recoveries of 50% and 75% were achieved 3 and 4 months after relapse, respectively, and a long-lasting response without relapse was obtained for at least 3 years.

**Conclusion:**

A combination of multimodal traditional therapies, including JKM formulas, acupuncture, and self-administered pine-needle stimulation, represents an effective integrative treatment for patients with AA.

## 1 Introduction

Sociopsychological distress is well-recognized today and remains a major burden for patients with alopecia.^[1]^ Alopecia areata (AA) is a condition of self-limiting, non-scarring hair loss. However, a significant number of affected individuals progress to chronic AA, which occasionally progresses into the severe forms of alopecia totalis and alopecia universalis.^[1,2]^ An underlying mechanism of AA that has been proposed is the local disruption of immune privilege within follicles,^[3]^ with topical corticosteroids and immunotherapy extensively used for limited lesions.^[1]^ However, a meta-analysis revealed that no treatments are currently available that are highly successful.^[4]^ Furthermore, the response of AA to treatment is unpredictable because recurrent exacerbations can occur, even during a successful course.^[1]^ Therefore, the unresolved pathogenesis and unpredictable treatment course of AA can cause considerable patient discomfort and morbidity.

Traditional Japanese Kampo medicine (JKM), which differs from traditional Chinese medicine in its origin and distinct historical development, is currently being integrated into modern medicine.^[5–7]^ East Asian traditional medicine including JKM has been demonstrated to successfully treat patients with AA using herbal formulas, acupuncture, and moxibustion.^[8–10]^ However, Japanese guidelines for the treatment of AA do not recommend JKM formulas nor acupuncture owing to a lack of solid evidence.^[11]^ To date, AA treatment consisting of multimodal traditional therapy combined with modern medicine has not been reported.

In this article, we report a young adult who presented with progressive and recurrent hair loss and was diagnosed with AA. He received a combination of JKM formulas, acupuncture, and immunotherapy. After an episode of relapse, a successful clinical outcome was achieved and maintained to date. Here, we describe pine-needle acupuncture that possibly enhances microcirculation of hairless lesions.

## 2 Case report

**Figure 1:**
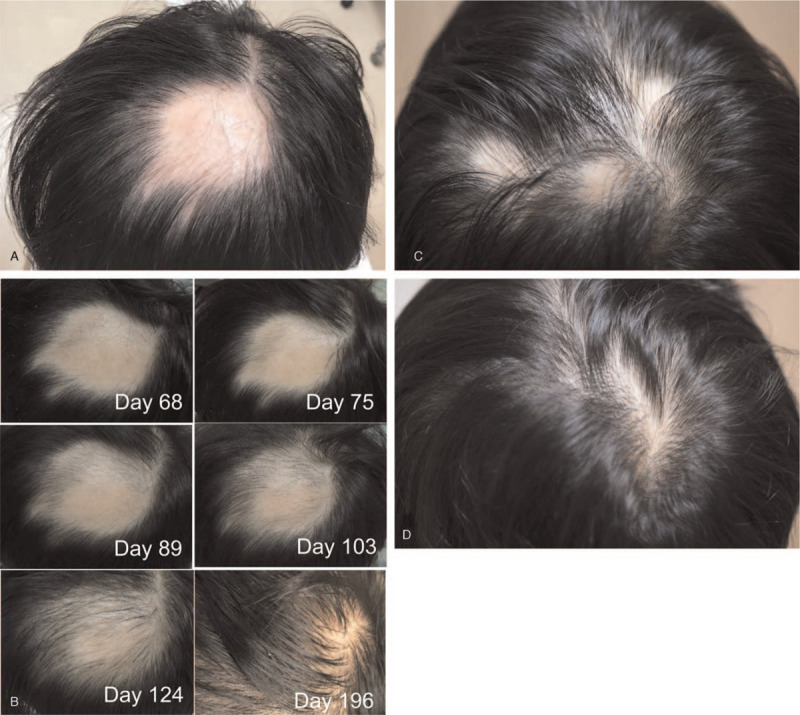
Sequential images of patchy hair loss. (A) A hairless 5 cm × 6 cm patch with residual exclamation mark hairs at diagnosis. (B) Sequential images are shown on day 68, 75, 89, 103, 124, and 196. (C and D) Multiple hairless patches at relapse on day 337 (C), which completely regrew hair on day 561 (D).

**Figure 2:**
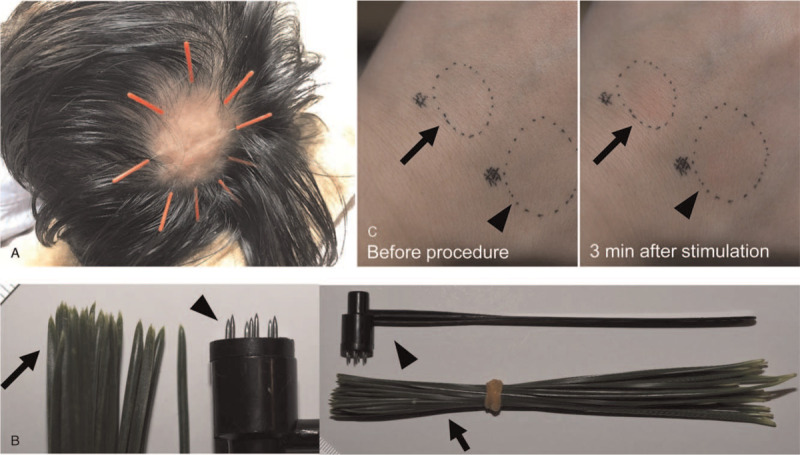
Acupuncture for alopecia areata. (A) Filiform needles (with a diameter of 0.16 mm) were inserted 1 *sun* (3.03 cm) laterally from the edge of the hairless patch toward the center and retained for 15 minutes. This acupuncture was performed weekly. (B) Comparison of self-administered pine-needle acupuncture (arrow) and disposable plum-blossom acupuncture (arrowhead). (C) Experimental stimulation on the hand dorsum by pine needles (arrow) and plum-blossom acupuncture (arrowhead). Stimulation was performed for 3 minutes. Note that both pine needles and plum-blossom acupuncture-induced erythema (right).

A 34-year-old male with a history of childhood asthma presented with a sudden loss of hair at the top of his head without any preceding symptoms (day 1). His general appearance was otherwise good, except for a hairless patch of 5 cm × 6 cm with residual exclamation mark hairs at its margin (Fig. 1A). He had no noticeable nail lesions or hairless patches elsewhere. He was thus diagnosed with AA with a single lesion (grade S1). He visited a dermatologist on day 5 and received liquid nitrogen stimulation weekly and squaric acid dibutylester treatment daily.^[12]^ However, he noticed an exacerbation of his hairless lesion, which urged him to consult the Kampo branch at the department of general medicine on day 7. He had been under a stressful situation at his workplace for 3 months before the onset of AA, leaving him with feelings of rage. Upon Kampo examination, he displayed the following symptoms: face redness, lack of sleep (<6 h/day), thirst, hot flashes, and excessive perspiration. The tongue diagnosis revealed red tongue coloration and blood stasis at the edge of the tongue with a thin and yellow coating. The pulse diagnosis revealed string-like smooth pulses that were sunken and weak at the cubit. Abdominal examination revealed significant strong abdominal tension, epigastric fullness/distension, fullness in the chest and hypochondrium, and slight weakness of the lower abdominal region. Collectively, the Kampo diagnosis was consistent with liver-qi stagnation and liver-yang rising. Pharmaceutical-grade saikokaryukotsuboreito^[13]^ extract (fine granules; 7.5 g/day; Kotaro Pharmaceutical Co., Ltd., Osaka, Japan) and rokumigan^[13]^ (granules; 7.5 g/day; Tsumura and Co., Tokyo, Japan) were administered. Filiform needles (J-Type, with a diameter of 0.16 mm, SEIRIN Corporation, Shizuoka, Japan) were inserted 1 *sun* (3.03 cm) laterally from the edge of the lesion toward the center and were retained for 15 minutes weekly (Fig. 2A). Local stimulation using plum-blossom acupuncture (Huan Qiu, Disposable Seven-star Needle, Suzhou Acupuncture Goods Co., Ltd., Suzhou, China) for 2 to 3 minutes once or twice a week was initiated as an adjunctive local therapy (see Video, Supplemental Video 1 [Supplemental Video 1 Exemplary plum-blossom acupuncture], which demonstrates the exemplary plum-blossom acupuncture technique). The Severity of Alopecia Tool (SALT) score was used to evaluate the treatment response of the patient for alopecia.^[14]^ His baseline SALT score was 19%. No significant change was observed after initiating these treatments except for the formation of 2 new small satellite lesions (SALT score 21%). On day 42, rokumigan was switched to shichimotsukokato^[13]^ (7.5 g/day; Tsumura and Co.) to treat blood stagnation. This change ameliorated his hot flashes and systemic perspiration. On day 47, white regenerated hair suddenly appeared, which became black on day 61 (SALT score 18%). The subsequent SALT scores were 17%, 15%, 13%, and 9% (>50% recovery from baseline) on days 75, 89, 103, and 124, respectively. Sequential photographs of the scalp are shown in Figure 1B. Finally, he achieved complete resolution on day 159, and thus dermatological treatments and acupuncture were stopped.

However, on day 322, three hairless lesions suddenly appeared that were not present upon his first diagnosis (Fig. 1C, SALT score 13%). Three weeks before he noticed this relapse, he underwent a very stressful situation again. This time, he only visited the Kampo branch, and saikokaryukotsuboreito and shichimotsukokato administration was reinitiated. For local treatment, he provided informed consent in compliance with the Declaration of Helsinki on performing self-administered pine-needle acupuncture himself every other day (see Video, Supplemental Video 2 [Supplemental Video 2 Exemplary pine-needle acupuncture], which demonstrates the exemplary pine-needle acupuncture technique).

The leaves of Japanese black pine (*Pinus thunbergii* Parl) are available across all of Japan. These leaves were sanitized using ethanol 70% (w/v) and preserved at −30°C until use. A bundle of pine needles resembles the tips of the plum-blossom acupuncture needles (Fig. 2B), and its stimulation induces local hyperemia in a similar manner as plum-blossom needles. Experimental stimulation was performed on the dorsum of the hand by pine-needle and plum-blossom acupuncture (Fig. 2C). Both methods induced similar erythema after stimulation for 3 minutes. Surprisingly, hair growth on the hairless patches occurred much faster than the first time. This time, more pine-needle acupuncture-induced petechiae occurred as his hair regrew. The sequential SALT scores were 11%, 5% (>50% recovery from baseline at relapse), 4%, and 2% (>75% recovery) on days 392, 420, 448, and 490, respectively (see representative photograph on day 560, Fig. 1D). To date (April 2021), he is still in remission and receiving saikokaryukotsuboreito-based formulas. Of note, the stressful environment at his workplace has remained unchanged throughout the course of his treatment and remission.

Patient interventions were conducted in compliance with the ethical standards of the Ethics Committee of Nagoya University Graduate School of Medicine. However, formal ethics approval was not obtained because this report is limited to just 1 case. Written informed consent for publication of this case report was obtained from the patient.

## 3 Discussion

This report is the first to combine the multimodal traditional medicine of decoctions and acupuncture together with contemporary dermatology. This is also the first report to describe pine-needle acupuncture for recurrent AA. Although the first choice for treating refractory AA under current guidelines utilizes local corticosteroid injections,^[1]^ this method lacks strong evidence. Complementary treatments are frequently reported in clinical and experimental settings,^[9,15,16]^ and a few clinical studies in this regard have been performed.^[17]^ Although these clinical studies demonstrate encouraging results, the medical needs of patients are still largely unmet.

AA is a type of systemic autoimmune disorder in which the breakdown of immune privilege in hair follicles suppresses hair growth via perifollicular infiltration of antigen-presenting and CD4^+^ and CD8^+^ T cells as well as abnormal expression of major histocompatibility complex class I and II molecules.^[3]^ This contributes to aggregations of CD56^+^/NKG2D^+^ natural killer cells, driving AA.^[1,2]^ In an experimental model of AA,^[10]^ tumor necrosis factor-α induces the vacuolation of stromal cells, and the intralesional temperature is increased, which may reflect local inflammation. Additionally, decreased angiogenic activity is reported in AA lesions,^[2]^ and impaired superoxide dismutase activity is reported in AA pathogenesis. Together, these studies emphasize the role of the local vascular system and oxidative stress in the pathogenesis of AA.

Traditional East Asian medicine recognizes hair as “an extension of the blood, which is stored in the liver” and that “the essence of the kidney lies in the hair.”^[9]^ The JKM guideline for AA is thus focused on normalizing blood to nourish hair by means of eliminating blood stagnation and/or replenishing qi and the kidney. The Kampo formula saikokaryukotsuboreito has been the key drug for this theory.^[8]^ Rokumigan, which replenishes the kidney, has been considered to be the adjunctive formula for alopecia; however, shichimotsukokato, which suppresses rising of the liver qi and thus normalizes blood stagnation, seemed to enhance regeneration in the present case. Shichimotsukokato has been traditionally administered to patients with renal hypertension. In a cellular model of kidney epithelium, ingredients from shichimotsukokato enhanced dimethylarginine dimethylaminohydrolase 2 and nitric oxide synthase, which may induce vasodilation by increasing nitric oxide.^[18]^ Our data on the effects of shichimotsukokato on cardiovascular dysfunction may shed light on the importance of improving local circulation for the treatment of AA.

Acupuncture has also been traditionally utilized in the treatment of AA.^[9]^ Electrical acupuncture enhanced hair recovery in a murine model of AA.^[19]^ Although several acupuncture points are known to contribute to hair growth,^[9,10]^ local stimulation using plum-blossom acupuncture irrespective of certain acupuncture points enhanced total hair regrowth in 58% of AA patients, whereas a recovery rate of only 34% was observed using topical 2% minoxidil treatment.^[15]^ In our case, we adopted self-administered pine-needle acupuncture at relapse. This is based on the anecdotal evidence from a 37-year-old female with AA, whose treatment was indicated by Masao Maruyama, MD^[20]^ (a talented Japanese acupuncturist who discovered the meridian phenomenon). This pine-needle acupuncture induced erythema similar to that induced by plum-blossom acupuncture. Interestingly, pine-needle stimulation did not induce microbleeding while hair growth was not observed by dermatoscopy; however, upon hair regrowth, petechiae appeared after stimulation, suggesting a recovery of local circulation at the lesion. The essential oil from *Pinus* spp contains terpenes, and its topical use induces local vasodilation. Volatile compounds from Japanese black pine needles exhibit antioxidant activities.^[21]^ The use of ethanol for sanitation may have coincidentally extracted these bioactive compounds from pine needles. Japanese black pine needles are also known to cause contact dermatitis, suggesting an effect on immunity.^[22]^ Although the role of local stimulation to enhance microcirculation has yet to be determined in clinical trials, pine-needle acupuncture is promising owing to the safety and availability of the procedure.

In conclusion, multimodal traditional treatment successfully and completely resolved AA. Given the limited number of treatment choices for AA, our findings are important, as they suggest that combined therapy using herbal formulas and acupuncture is a potential strategy that warrants further research.

## Acknowledgments

The authors thank Dr. Naoya Goto, MD, PhD, from the Department of Dermatology at Nakatsugawa Municipal General Hospital for providing clinical photographs.

## Author contributions

**Conceptualization:** Nozomu Kawashima, Xiaochen Hu, Takaharu Matsuhisa, Juichi Sato.

**Data curation:** Nozomu Kawashima, Takaharu Matsuhisa, and Juichi Sato

**Funding acquisition:** Nozomu Kawashima

**Resources:** Xiaochen Hu, Nagako Ishikawa, and Takaharu Matsuhisa

**Supervision:** Juichi Sato

**Writing – original draft:** Nozomu Kawashima, Takaharu Matsuhisa, and Juichi Sato

**Writing – review & editing:** Nozomu Kawashima, Xiaochen Hu, Nagako Ishikawa, Takaharu Matsuhisa, Juichi Sato.

## Supplementary Material

Supplemental Digital Content

## Supplementary Material

Supplemental Digital Content
